# Emerging Food Fortification and Precision Nutrition: Technologies for Personalized Nutrient Delivery and Health Monitoring

**DOI:** 10.3390/foods15213823

**Published:** 2026-10-26

**Authors:** Fahad Saad Alhodieb

**Affiliations:** Department of Basic Health Sciences, College of Applied Medical Sciences, Qassim University, Buraydah 51452, Saudi Arabia; f.alhodieb@qu.edu.sa

**Keywords:** encapsulation technology, food fortification, nutrition, precision nutrition, public health, bioavailability, functional foods

## Abstract

Food fortification has experienced a revolution because of the process of encapsulation techniques, controlled delivery systems, and personalized nutrition approach. The purpose of this review is to analyze the recent trends in encapsulated and personalized food fortification systems and the possibility of increasing the stability, bioavailability, targeted delivery, and personalized nature of nutrients. A review of the scientific literature was performed, covering topics such as micro- and nanoencapsulation, microfluidization, emulsified systems, omics approaches, nutrigenomics, nutrigenetics, microbiome interventions, 3D/4D food printing technology, wearable biosensors, artificial intelligence dietary analysis, and Internet of Things in foods. According to the reviewed literature, these technological approaches could help protect, distribute, and release vitamins, minerals, probiotics, and bioactive compounds, and help provide the opportunity to develop personalized nutritional approaches. Moreover, integration of omics techniques with advanced food technologies would allow taking into account genetic, metabolic, and microbiome aspects of different nutritional needs. Furthermore, emerging digital and manufacturing technologies would also provide an opportunity to create individualized fortified foods. Nevertheless, there are still issues with regulatory aspects, cost-effectiveness, consumer acceptability, and the equity and privacy of genetic and personal data. In conclusion, the application of controlled-release technology in personalized nutrition techniques holds much promise in creating specialized fortified foods, while additional research is required to establish their safety, effectiveness, affordability, and practical application.

## 1. Introduction

The World Health Organization (WHO) and Food and Agriculture Organization (FAO) have recognized food fortification as a sustainable solution and, therefore, it has become an international strategy in addressing the deficiencies of micronutrients, like iron, iodine, vitamin A, folic acid, and zinc [1]. Universal Salt Iodisation (USI) was effective in minimizing the number of iodine deficiency-related diseases because it ensured that iodised salt was widely used and thus helped in the prevention of intellectual disability that is prevalent when there is a lack of iodine [2]. Fortification has also been effectively used to address other shortages; iron fortification of staple foods has reduced the incidence of anaemia amongst vulnerable populations like women and children [3]. However, problems such as disproportional distribution in rural areas and the presence of dietary inhibitors highlight the need for additional policy support and technical advances [4]. Countries that have implemented folic acid fortification have reduced cases of neural tube abnormalities, although there has not been uniform implementation, as is the case with iodisation. Thus, although food fortification has been found to have positive effects on public health, the initiative must address the real-world challenges so that its effects on micronutrient deficiencies worldwide can be maximized [5]. The significant place of gut microbiota and gene-diet interactions in dietary effects and health outcomes can be used as an exemplar of the changing paradigm of population-based nutrition to personalized nutrition [6]. Current studies emphasise the effects of the gut microbiome on metabolic diseases like metabolic syndrome, type 2 diabetes (T2D), and cardiovascular diseases (CVD) because of their diversity and composition [7]. The need to use personalized dietary recommendations can be explained by the fact that, in the case of, say, a study that was based on the use of gut microbiota fingerprinting, it has been shown that the ability to predict the glycemic reaction to meals can vary significantly across people [8]. This change is also demonstrated by the impact of the genetics of the host on the microbiota of the gut, as some genetic differences can influence the response of the gut microbiome to dietary cues [9]. Besides, there are population-based studies that propose that individualised dietary measures based on individual histories may lead to better health. The PREDIMED trial evaluating compliance with the Mediterranean diet is one such study. This more sophisticated method of nutrition makes precision nutrition methods possible because it considers both genetic and individual microbiome profiles [10].

Nutrigenomics explores the interactions between the components of foods and the genome and provides a foundation for identifying how genetic differences might impact the metabolism of nutrients and health effects [11]. Nutrigenetics aims to understand how variations in genes can affect the response to nutrients, while nutri-epigenetics identifies how diet influences the expression of genes through epigenetic mechanisms [12]. Furthermore, there are transcriptomics, proteomics, and metabolomics contribute information related to gene expression, protein response, and metabolism due to the consumption of foods [11]. Keeping this in mind, the present review emphasizes the historical development of food fortification from traditional population-based strategies to more innovative and personalized methods [13]. We discussed the historical development and limitations of traditional food fortification and the development of precision nutrition, with emphasis on nutrigenomics, nutrigenetics, epigenetic modulation, and omics tools. We also highlight the new advancements in personalized food fortification using artificial intelligence (AI) or machine learning (ML) algorithms, smart delivery systems, nano/micro encapsulation, 3D/4D printing of food, lab-on-a-chip technologies, wearable biosensors, and the Internet of Things (IoT) [14]. In particular, we also emphasize personalized fortified foods, functional ingredients, and the role of the gut microbiome in nutrient metabolism. Clinical evidence for new methods is evaluated, as well as the ethical, regulatory, and safety concerns associated with the development of fortification technologies. Eventually, our review highlights major economic, technical, regulatory, and consumer issues for the fortification technology development. Moreover, this review emphasizes the possible research directions in the area of personalized fortification technologies to support safe, efficient, sustainable, and equitable nutrition and public health.

### Literature Study

A systematic literature search was performed in PubMed, Scopus, Web of Science, and Google Scholar using publications from 2015 to 2026, with older landmark publications being considered where necessary. Keywords used include ‘food fortification’, ‘biofortification’, ‘functional foods’, ‘nutrient delivery’, ‘encapsulation’, ‘personalized nutrition’, ‘nutrigenomics’, ‘gut microbiome’, and ‘precision agriculture ’, and were connected using Boolean operators AND/OR. Original research, clinical trials, observational studies, technological studies, and review articles were considered in the search, with any duplicate, off-topic, and irrelevant publications being discarded. The evidence was classified as clinical, observational, experimental/technological based on reviews, or future or emerging applications in order to ensure proper interpretation of the claims made [15,16].

## 2. Evolution of Food Fortification: From Conventional to Personalized Approaches

**Figure 1:**
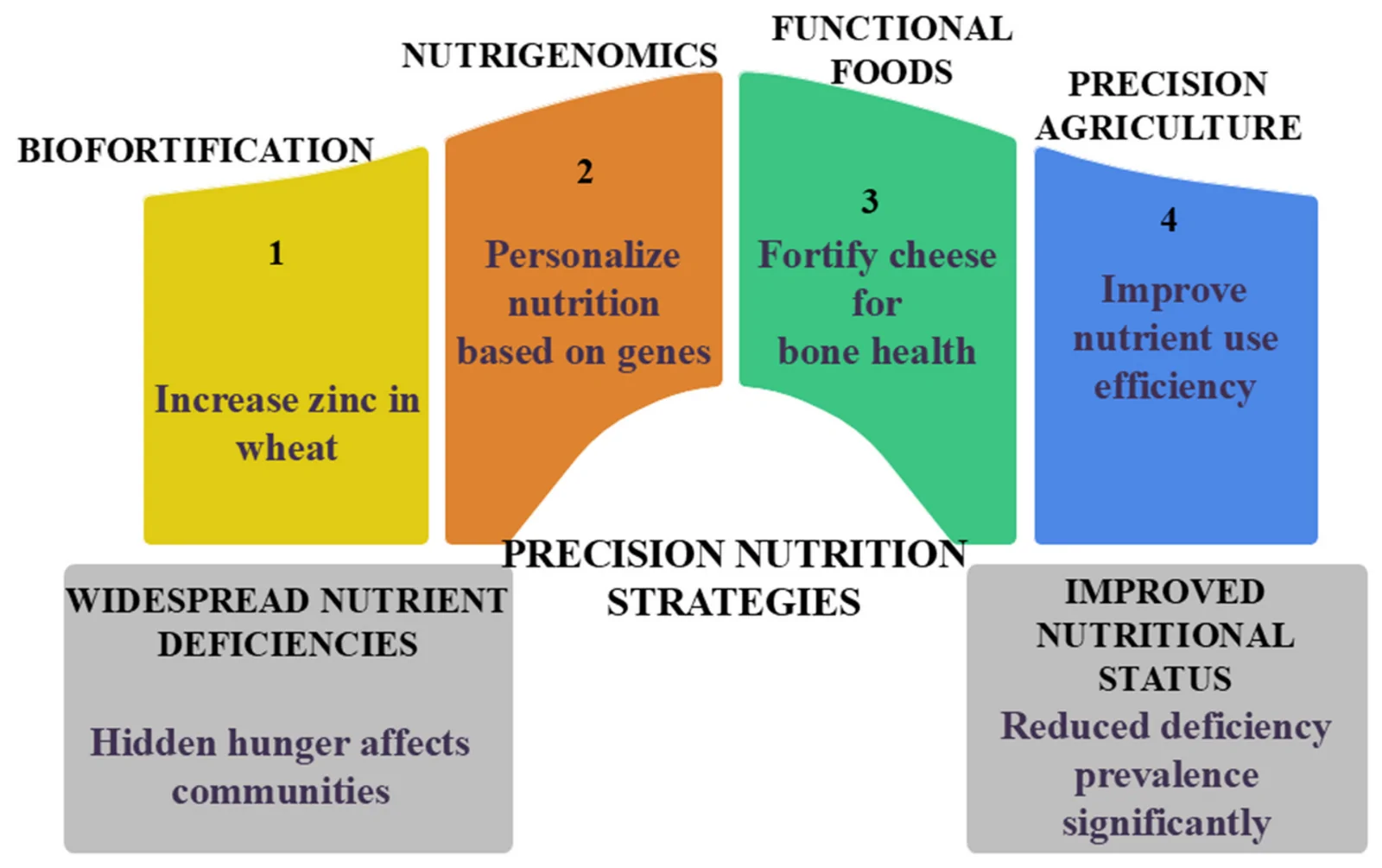
Improving nutrition through precision methods.

Landraces with high zinc content were hybridised with high-yielding wheat to produce cultivars that contain 50 per cent more zinc than normal wheat [17]. An Indian study carried out a randomized controlled trial on zinc-biofortified and zinc post-harvest fortified wheat flour added to school lunches for children. The addition of zinc-enriched wheat flour did not show any improvement in zinc levels in plasma despite increasing zinc intake [18]. These findings demonstrate how biofortification can be used as a population-level precision nutrition strategy by increasing the concentration of essential micronutrients in commonly consumed staple foods. The most promising expertise in individual nutrition strategies is nutrigenomics at the forefront. A case study [19] explains genetic testing to identify polymorphisms in genes such as MTHFR, VDR, and BCMO1 that influence nutritional metabolism and requirements. In addition to one-size-fits-all approaches, personalized prescriptions of vitamins and minerals are developed based on the unique genetic predispositions and dietary habits in nutrition [20]. Thus, nutrigenomics represents a precision approach that can help tailor nutritional recommendations according to individual biological characteristics, as illustrated in Figure 1. Some of the clinical investigations of vitamin K2-containing cheese have suggested alteration in the markers related to bones [21]. In one clinical study involving a randomized trial of Jarlsberg cheese consumption per day, it is noted that there is an increase in the levels of osteocalcin as well as PINP with no significant change in CTX [22]. These results suggest alterations in bone turnover markers but not increase in bone mineral density. Clinical trials showed improved bone mineral density, and decreased markers of bone turnover were observed relative to unfortified cheese. This example illustrates how functional foods can be specifically formulated to deliver targeted nutrients and provide health benefits beyond basic nutrient requirements. Precision agriculture platforms are also an example showing integration of methodology and technology to improve nutrient use efficiency and nutrient management overall, and precision in crop production systems with emphasis on data analysis, remote sensing, and ML technologies [23] (Figure 1). Precision farming employes scientific methodologies like decision support systems and ML techniques for efficient nutrient use. During trials conducted in rice, wheat, and maize cultivation in the Eastern Indo-Gangetic Plains, the use of nutrient expert advice coupled with ML techniques was more effective in improving crop production than farmer practices [23]. As shown in Figure 1, precision agriculture complements biofortification and precision nutrition by improving the efficiency of nutrient application and crop production, thereby supporting the sustainable production of nutrient-rich foods. Figure 1 shows how biofortification, nutrigenomics, functional foods, and precision agriculture form a set of interrelated precision nutrition techniques. The former involves nutrient density enhancement of staple crops, while the latter involves personalized nutrition using information about one’s genome and diet. Functional foods involve delivering health-beneficial nutrients via enriched food products. Precision agriculture is aimed at enhancing nutrient use efficiency in crop production. Overall, these four methods combined can play a role in addressing the problem of malnutrition and hidden hunger. The above-discussed examples show how a transition occurs from the mere delivery of nutrients to more complex methods including genetics, food technology, biotechnology, and precision agriculture. Fortification programs involving folic acid, vitamin D, iodine, and iron supplementation have made a valuable contribution to people’s well-being [24,25]. Thus, for instance, regular consumption of iodized salt has contributed significantly to the prevention of iodine deficiency, as 86% of households consume this kind of salt [26].

### 2.1. Limitations of Conventional Fortification Models

Food fortification faces its own challenges and issues, particularly concerns with over-fortification and consumer acceptance of fortified foods. Consumption of excess nutrients may cause health issues, such as vitamin toxicity or gastrointestinal disturbance due to higher intake of iron [27,28]. In cases of mandatory fortification, the nutritional needs of a population may not be considered, putting individuals at risk for significant illness and excluding individuals who could use some support [29]. Confusion or distrust among consumers about fortified foods may include association of food fortification with negative characteristics, such as unnatural and unappealing, and impair acceptance of fortified items [30]. Added nutrients may negatively affect the sensory perception and desirability associated with foods [31]. Each of these factors brings some attention to the potential barriers and challenges of traditional food fortification programming, which need to be addressed to develop the most effective interventions.

### 2.2. Emergence of Precision Nutrition and Data-Driven Food Design

A defining feature of precision nutrition’s emergence is the meaningful advances in data-driven approaches to optimising individual health with personalized dietary interventions [32]. A primary trend in this movement has to do with the increasing sophistication of online platforms that deliver behaviour change mechanisms and feedback, informed by the scientific literature, for interventions. Novel strategies, including ML algorithms and monitoring glucose regularly, have allowed scientists to identify individual variability in metabolic responses [33]. Metabolic phenotyping can assess glycemic responses to food intake, and in a case study, support personalized dietary recommendations that optimise health behaviour [34]. Consequently, precision nutrition could transform public health models by providing individuals with personalized and timely information that encourages improved health behaviours, beginning with education [35]. This model is illustrative of how interdisciplinary models may embed health data into learning environments, helping develop a proficient public health population in precision health behaviours.

Utilising personalized health data to enhance food fortification solutions necessitates the amalgamation of omics methodologies; in particular, multi-omics like transcriptomics, proteomics, metabolomics, and genomic sequencing [36]. Practitioners will be able to create more tailored nutritional therapies by investigating the interaction between each of the omics layers and how lifestyle and nutrition impact each layer. The application of multi-omics methods to individual profiles is useful for the identification of metabolic dysfunction and health risk [36]. Early identification of health issues is beneficial for both preventative measures, such as dietary changes and personalized interventions, because of being able to apply this type of personalized profiling [37]. Circadian rhythms can influence therapeutic responses and health outcomes; the inclusion of circadian rhythms into the multi-omic analysis is further additive and deepens the understanding of how personal health data can help inform effective food fortification programs [38] (Figure 1).

### 2.3. Nutrigenomics and Nutrigenetics in Personalized Nutrition

The investigation of the role of genetic variation in the response to nutrients and the implications for personalized nutrition is referred to as nutrigenomics and nutrigenetics [11]. Several studies have offered promising evidence of significant nutrient-gene interactions, yet their clinical relevance remains limited. For instance, several studies identified that certain genotypes might modify fatty acid metabolism or increase the risk of nutritional deficiencies [39]. However, the strength of the association with health outcomes is not at the level that would support personalized dietary recommendations at this time [40]. In addition, there is insufficient evidence that findings from any one ethnic group could extend to others, which requires caution in application to a few ethnicities [41]. Integrating genetic data in dietetic practice is further complicated by limitations such as the methodological flaws from previous research and the absence of training to interpret genetic data by health professionals [42]. Gene expression analyses have not demonstrated the ability to inform personalized nutrition, and the applicability of results will depend on validation from clinically relevant biological specimens [43]. Based on nutrigenomics, nutrients have an important part in gene expression through numerous mechanisms [11]. As signalling molecules that regulate the expression of genes, proteins, and metabolic pathways in humans, the chemicals present in ingested food can either have a primary influence on gene expression directly or indirectly [44,45,46].

Studies of eicosapentaenoic acid and docosahexaenoic acid-comprising supplements have also demonstrated examples of a genomic effect on treatment efficacy, with some dependence on sex. These results accentuate the importance of understanding how genes and diet interact to develop personalized dietary recommendations that optimise health outcomes, in accordance with a person’s genetic factors [47,48,49]. The significance of understanding gene-nutrient connections, specifically the relationship between gene functions and the consumption of nutrients, cannot be overstated and is important for comprehending health disorders [50]. MTHFR produces an enzyme involved in folate-dependent activities, including DNA synthesis and methylation [51]. Folate consumption is critical before conception, during pregnancy, and throughout life, especially for those with MTHFR polymorphisms, since variation in the MTHFR gene appears to significantly affect folate metabolism. A high dietary folate intake, for example, can modify and diminish health risks complicated by these gene variations in the presence of diseases such as homocysteinuria [52]. Conversely, the APOE gene affects brain and cardiovascular health and is closely associated with lipid metabolism. Genetic variation for the APOE gene (APOE ε2 & ε4) modifies lipid metabolism in the body, and it is linked with an increased risk of disease, including schizophrenia, in some patient populations. Specifically, changes in lipid levels may interface with gene expression, such as findings reporting links between elevated APOE alleles (e.g., ε2) and increased risk of illness for some patient populations [53,54].

#### 2.3.1. Influence of Genetic Polymorphisms on Micronutrient Necessities

**Figure 2:**
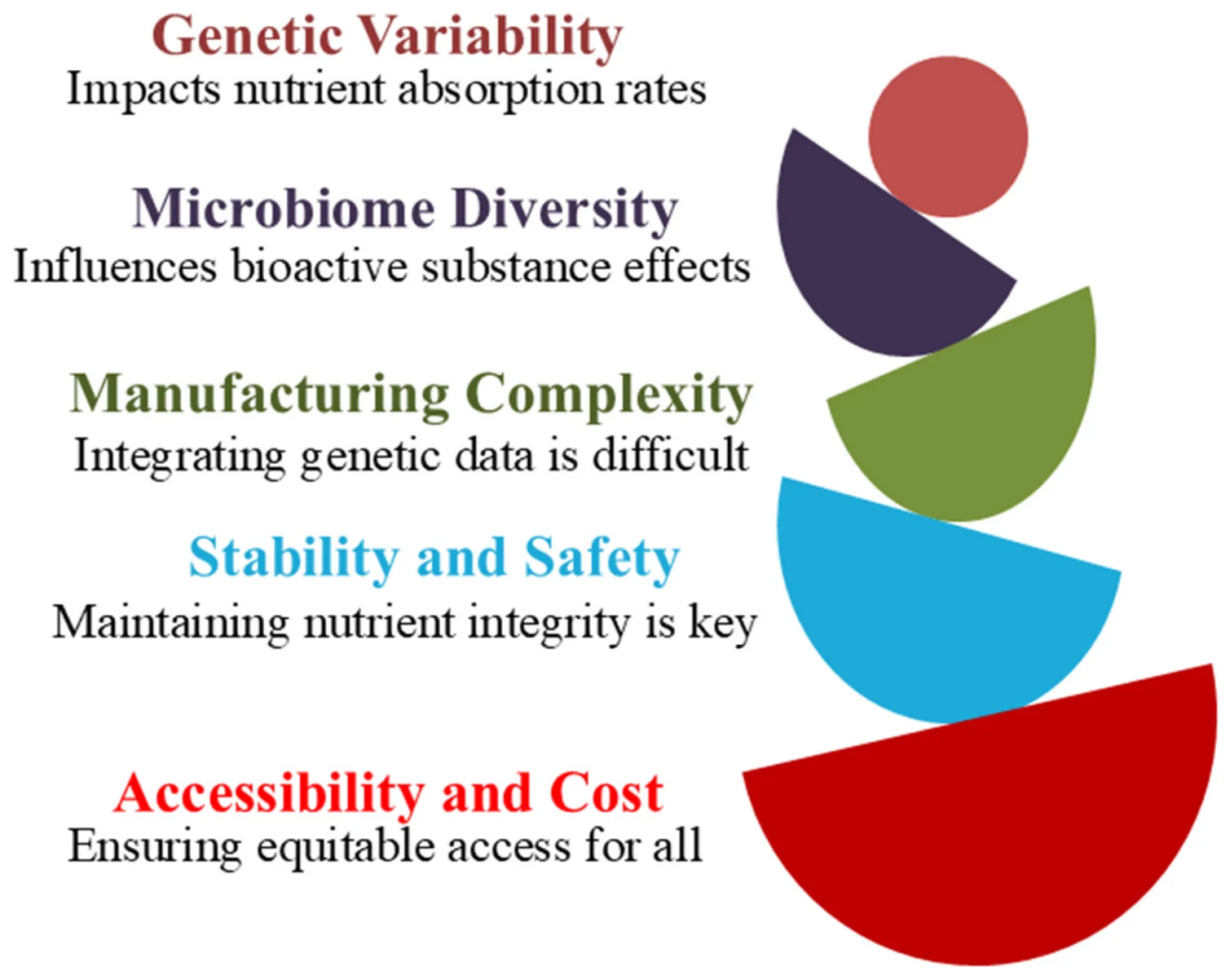
Personalized fortified foods: Overcoming design challenges.

Genetic polymorphisms markedly influence the metabolism and necessity of micronutrients in human nutrition, primarily with genetic polymorphisms affecting iron and vitamins D, B12, folate, and thiamine. The absorption, transportation, and metabolism of all micronutrients, including some genes that have been specifically studied with specific micronutrients, modify the absorption, transport, and overall metabolism within the body [55]. To illustrate, polymorphisms in the FUT2 and CUBN genes are critical for vitamin B12 absorption, and polymorphisms in the GC, CYP2R1, DHCR7, CYP24A, and VDR genes are factors in vitamin D status [56]. Likewise, individuals who may be at risk for low folate can be identified with carrier status for the MTHFR genetic polymorphism. Genetic variability in transport proteins for iron and thiamine is another example of how genetic variations can result in deficiencies or altered nutritional needs. Thus, understanding these factors is important for modifying dietary interventions to manage and prevent deficiencies [57]. Figure 2 shows the various obstacles that arise in the development of personalized fortified foods. Nutrient uptake and individual nutritional needs may vary due to genetic variations, while variation in microbiome diversity in the gut may affect the metabolism and effects of nutrients and bioactive components. Converting individualized nutrition data into functional food products poses its own set of manufacturing problems, including consistency in formulation and quality of the end product. Moreover, the stability, bioavailability, and safety of the nutrients and their effects should be ensured during processing and storage of these personalized fortified foods. The accessibility and cost of the process are also crucial to consider to ensure that such personalized fortified foods can be applied on a large scale.

#### 2.3.2. Epigenetic Regulation and Its Implications for Long-Term Dietary Adaptation

The phrase “epigenetic regulation” refers to inheritable alterations in gene expression that occur without any changes to the DNA sequence, and it is mainly affected by various factors, including diet [58]. Subsequently, long-term health outcomes are intensely influenced by dietary factors through epigenetic regulation. Both macro- and micronutrients from the diet can change epigenetic marks like DNA methylation and histone modifications [59]. These modifications play a vital part in regulating gene expression, thus shaping an individual’s susceptibility to chronic disorders. The epigenetic mode of action is highly responsive to environmental cues, specifically dietary patterns prominence the intricate interplay among nutrition and health across the lifespan [60]. Hence, ensuring optimal nutrition during crucial phases of development is essential for decreasing the risk of chronic disorders later in life, underlining the significance of targeted nutritional interventions for all populations [4].

### 2.4. Omics Technologies Driving Precision Fortification

Genomics-based technologies play a significant role in advancing nutrition science through their key branches like transcriptomics, epigenetics, and nutrigenomics. Nutrigenomics explores how nutrients influence gene expression, resulting in changes in protein synthesis and metabolite production, thereby shaping individual health outcomes. Moreover, transcriptomics focuses on analyzing the complete set of RNA transcripts to understand how diet, exercise, and other factors affect gene expression, which results in influencing disease susceptibility. On the other hand, epigenetics examines the reversible variations in gene activity that are influenced by environmental and nutritional factors, pinpointing the dynamic relationship between diet and health. Altogether, these genomic methods improve personalized nutrition by providing a deeper understanding of individual nutritional requirements that depend on genetic and epigenetic profiles. Within this context, transcriptomics holds specific significance in nutrigenomics through gene expression microarray technology [61]. This technique allows simultaneous assessment of thousands of genes, enabling a comprehensive study of nutrient-gene interactions [62]. Consequently, it helps scientists to determine the biological impacts of dietary components on gene expression and assess the overall influence of nutrition through the nutrition-related transcriptome [63]. Microarrays have been used to identify the genes expressed differently depending on a dietary condition, and to identify targets to provide potential markers for diagnosis and prognosis of diseases [64]. Notwithstanding the improvements from advances in technology, scientists still deal with technical variation and need to continue being cautious to ensure accurate collection and interpretation of data [65,66,67]. The focus of metabolomics is to understand how different environmental stimuli, including nutrition, relate to the response in metabolism [68]. Metabolomics allows researchers to study groups of metabolites in the context of specific metabolic pathways, yielding information about the potential impacts of dietary components on health and disease [69]. This has been regarded as the method of choice for human molecular analysis of food biomarkers, dietary patterns, and food consumption. Dietary pattern studies that look at the physiological effects of chocolate, coffee, and fiber consumption demonstrate that metabolomics can be used to assess the mechanism by which human beings respond to dietary intake [70]. Dietary methods and/or dietary pattern studies have been supported by advances in analytical technology, but still represent limitations to a complete measurement and identification of all metabolites within the human body [71].

#### AI, ML, and Big Data Analytics for Personalized Diet Prediction and Fortification Planning

AI, ML, and big data analytics are transforming personalized dietary prediction and fortification planning through a variety of strategies [72,73,74,75]. The use of genetic data and gut microbiota to make personalized nutrition recommendations matched to individual metabolic response and health needs is an exciting application of these technologies [76]. For instance, AI can recognize genetic correlative predispositions to employ personalized dietary programs, reducing the risk of disorders like diabetes and obesity’s prevalence [77]. Additionally, AI technologies provide personalized dietary recommendations based on the information obtained from wearables and mobile health applications that measure lifestyle variables, namely, stress and physical activity, providing real-time, dynamic dietary recommendations based on the individual’s current state [78]. The use of omics technologies offers important information about the individual’s genetic, metabolic, and physiological profile, hence allowing the assessment of their nutritional needs. Nonetheless, the conversion of such molecular information into practical strategies for fortifying diets necessitates the use of appropriate technological systems to formulate, deliver, monitor, and adjust doses. Hence, the incorporation of information obtained from omics technologies with new food technologies is an important link between understanding nutritional needs and developing personalized fortified diets.

### 2.5. Technological Innovations in Personalized Food Fortification Science Identifiers

Smart fortification systems incorporate advanced technologies, including smart nano-delivery platforms, which will potentially release nutraceuticals adaptively to satisfy the nutrient demands of an individual. Smart fortification systems ensure that nutrients are delivered to the body when and where they are needed by responding to internal physiological signals, such as pH, temperature, and enzyme level [79,80,81,82]. For example, wearable glucose biosensors have the potential to improve overall nutrition management by influencing food choices, in addition to being essential for managing diabetes [83]. To provide timely and individualized suggestions for improved dietary adherence, recent technologies have shown the potential to detect nutrient levels, such as vitamin C in sweat [84]. By offering insights on food intake and metabolic status, this novel technique facilitates adaptive nutrient delivery depending on individual needs [85]. Numerous clinical applications and health effects have been established by smart fortification systems that use targeted and adaptive nutrition delivery [86]. For example, ubiquitous salt iodisation has saved almost 750 million cases of iodine deficiency disorder (IDD) and decreased goitre rates by 86% worldwide [87]. Neural tube abnormalities have also significantly decreased because of folic acid fortification of rice and wheat flour; the program prevents about 22,000 instances each year in the United States [88]. Furthermore, vitamin D fortification has successfully decreased rickets in at-risk populations by 70% in North America, Europe, and the Middle East [89,90,91]. Although these advanced technologies allow for the detection, prediction, and monitoring of individual nutrient needs, the successful implementation of personalized fortification relies on the delivery of those nutrients in a stable, bioavailable, and controlled manner. In this regard, encapsulation technologies offer an important technological bridge connecting personalized nutrition and personalized delivery of selected nutrients. Encapsulation technologies have the potential to help bridge personalized nutritional goals and food products.

## 3. Nano- and Microencapsulation Technologies for Controlled Nutrient Release

The development of encapsulation techniques has opened up new possibilities for increasing the stability, bioavailability, and controlled release of nutrients and bioactive compounds in fortified and functional foods [91]. Encapsulation processes include embedding sensitive nutrients using protective matrices to decrease their exposure to external conditions like oxygen, light, water, temperature, and interactions with other food components. Thus, encapsulation helps prevent nutrient degradation during food processing and storage and allows for their controlled release during digestion. The development of nutrient delivery systems can involve the use of various edible coating agents such as chitosan, alginate, proteins, lipids, starches, and other biodegradable polymers [92]. The process of microencapsulation would be helpful in providing protection against the degradation of vitamins, minerals, polyphenols, omega-3 fatty acids, and probiotics, while the method of nanoencapsulation could be more beneficial due to an increase in the surface area, dispersibility, and possible improvement in the bioavailability of the poorly soluble nutrients. This technique would be highly important for fortified foods. In addition to protecting nutrients against the adverse effects of processing and storage, controlled release can allow nutrients to be delivered in a targeted fashion through different phases of the process of GI digestion, resulting in enhanced accessibility and efficacy of nutrient absorption. Encapsulation may also be helpful to hide any unpleasant taste, odor, or coloration from certain micronutrients and phytochemicals. Therefore, nano- and microencapsulation technologies offer promising approaches for circumventing drawbacks inherent to traditional nutrient fortification methods [93].

### 3.1. 3D and 4D Food Printing

Food structure and nutrient composition can be controlled with 3D and 4D food printing [94]. 3D printing allows for precise control over food composition and structure, as well as for making personalized food products that consider dietary differences associated with age, gender, or weight [95]. It also enables the production of food products with novel microstructure to regulate nutrient release and absorption from the digestive system to improve nutritional outcomes for consumers. In addition, it meets nutrition needs without food waste, which is an important consideration for sustainability [96]. Recent studies have shown how 3D printing technology can be used for nutrient fortification and customization of food products. A study by Lim et al. used cholecalciferol (Vitamin D3) loaded Pickering emulsion with the help of a complex formed by pea protein isolate and inulin to create 3D-printed food products. It displayed excellent dispersion stability and slow lipid digestion, indicating the utility of 3D printing technology in making vitamin D-fortified foods [97]. Recently, another study fabricated 3D-printed carrot foods supplemented with riboflavin-enriched whey protein isolate nanoparticles. The addition of the nanoparticles enhanced the bio accessibility of riboflavin by 23.1% under in vitro digestion, thereby revealing that 3D printing can be coupled with nano-delivery systems to increase the nutritional properties of printed foods [98]. In another study, an abalone-based 3D-printing ink containing soybean protein, polydextrose, vitamin C, and gellan gum was developed specifically for senior-friendly foods, illustrating the potential of 3D printing to simultaneously address nutritional and textural requirements of older consumers [99].

4D printing offers the same advantage, but extends it by enabling responsiveness to environmental cues and change over time, opening new platforms for producing foods with functional characteristics or generating custom foods to meet tailored nutrition [100]. Furthermore, a study on 4D food printing has revealed that foods made through printing technology are capable of changing color, taste, or shape under certain stimuli. For example, a 4D printed food consisting of anthocyanin-rich purple sweet potato puree mixed with mashed potatoes has been created, which changed colors spontaneously under the influence of pH changes [101]. Recently, the development of 4D printed foods through microwave-induced 4D printing of vegetable-based foods has enabled the possibility of programmable shape changes [102]. Furthermore, a 2026 research study by Xu et al. showed that it is possible to design 4D food using microwave-induced shape transformation of 4D printed pea-based dough, where printing path design, infill density, and baking process parameters can determine shape transformations [103]. These examples illustrate that the 3D and 4D printing of foods is advancing from conceptual applications to functional food systems where the nutrient content, bio accessibility, and structure can be purposefully designed for particular consumer demands.

### 3.2. Lab-on-Chip

The influence of lab-on-a-chip technologies on food safety, processing, and packaging has increasingly garnered attention [100]. There are two main limitations to commercializing lab-on-a-chip applications that need to be resolved before practical applications, mass-scale manufacture, and user acceptance [104]. Technologies are being developed to improve the efficiency of food monitoring practices and create new styles of fast and low-cost food safety and quality detection with small sample sizes [104]. Additionally, lab-on-a-chip technology is expected to enable end-to-end traceability of products along the food production chain, an essential component of real-time nutritional monitoring [100]. The creation of nano-encapsulated nutrient delivery systems is one of the most recent developments in lab-on-a-chip applications, demonstrating the variety of integrated strategies that are conceivable with this technology. More comprehensive solutions in food technology may potentially result from combining these devices with methods for the value-adding of food and agricultural waste [105].

The creation of decentralized self-testing devices that examine different nutrition-related metabolites and hormones, like lactate and glucose, is one recent development in biosensor-based systems for real-time nutritional monitoring [83]. These technologies provide users with insight into their nutritional status by enabling them to evaluate their status in the moment and substitute centralised tests that take longer with portable electrochemical detectors. In various portable devices, enzymes are used in disposable strips to generate rapid macronutrient assays [106]. Recent developments in health-related areas of personalized nutrition initiatives have incorporated wearable biosensors with specifications that encompass electrical components that could continuously monitor food and supplement intake at the molecular level [107]. Recently developed technologies have demonstrated the possibilities of real-time data collection within nutrition management, combining stretchable electronics and microstructured sensors to monitor sodium levels in real-time from a variety of food sources [107]. In the last decade, there have been tremendous advances in individualised nutrition using mobile and wearable chemical sensors [108]. However, technology and devices for monitoring nutrition using motion tracking, calorie-counting apps, etc., are still not able to provide molecular-level monitoring of nutrition. Non-invasive biosensors, which monitor chemical changes occurring in the body after food consumption, are now critical technologies for monitoring individualised nutrition [109]. Moreover, there are opportunities to merge these wearable chemical sensors with data analytics methods to improve the precision of nutrition monitoring with decision-making based on nutrition-related changes [110]. The development of these types of systems could produce an entirely new category of real-time nutritional monitoring solutions.

### 3.3. Digital Twins and IoT-Enabled Food Devices for Personalized Fortification at the Point of Consumption

Digital twins (DT) are innovative technologies that enable the simulation of many agricultural situations and practices for accurate forecasting and better management of crops. In a digital environment, DT allows farmers and experts to try out several strategies and consider potential outcomes [111]. With the use of IoT technologies, there is real-time monitoring and improved decision-making opportunities to augment farming and food supply chains. These IoT methods ultimately allow decisions to be made in real-time at the point of consumption, for example, tailoring fortification in food products, by reducing waste, increasing efficiency, and providing better visibility through logistical functions [112]. This study discusses the use of DT technology that leverages IoT and ML algorithms to create a digital copy of humans [113,114]. Evidence of tailored dietary interventions can be found in the helpful applications to dynamically evaluate the effects of foods on blood glucose levels available in this individualized mobile and web-based system [115]. The paper discusses the advancements of the IoT and how it can assist with tailored nutrition. It raises the potential of mobile and wearable chemical sensors that monitor food intake in relation to physical activity [116]. This means that personalized food fortification is not only the choice of the right nutrient dose but also its delivery in food systems. Encapsulation can make nutrients more stable, more available, and easier to release. Moreover, it allows for the reduction of negative sensory properties. Based on these features of delivery, the next section examines ways in which these technologies can be integrated into personalized food fortification.

### 3.4. Designing Personalized Fortified Foods

#### 3.4.1. Tailored Fortified Food Design

To enhance potential health benefits, personalized fortification may involve designing functional foods according to individual biological, genetic, metabolic, and dietary characteristics. However, the evidence for individualized foods is still early, and mechanistic or observational data cannot be equated to clinical efficacy. Genetic and microbial profiles can be used to uncover variation in nutrient metabolism or responsiveness, but correlation does not equate to causation, and these variations have yet to be proven capable of preventing disease or achieving better clinical outcomes through genotype- or microbiome-directed foods. For example, an individual who has the genetic variant called APOE4 may be advised to increase consumption of viscous fiber in their diet based on their metabolic status, while another person may be recommended a soy protein bar or yogurt fortified with sterol [117,118,119]. These examples represent potential applications rather than established clinical interventions. The evidence obtained from randomized controlled trials concerning personalized nutrition is more conclusive in the improvements that can be made in selected metabolic indicators. This evidence includes an RCT on 347 subjects where the outcome was the improvement of cardiometabolic factors in response to individualized dietary interventions that involved postprandial glucose and triglyceride responses, microbiome factors, and health history. Nonetheless, this RCT did not consider a personalized fortified food item, but just personalized dietary recommendations. Another piece of evidence comes from a 12-week RCT conducted on 400 obese Chinese adults where improvements were recorded in BMI, body fat percentage, waist circumference, and some blood biomarkers through a personalized nutrition intervention. This gives more credence to personalized nutrition and food fortification but does not necessarily mean that personalized nutrition and food fortification are proven [120,121]. As such, the current state of evidence on personalized fortified foods is not as robust as that on standard fortification programs. To establish whether personalized fortified foods provide additional benefit to standard fortification programs, well-conducted randomized controlled clinical trials will have to be carried out.

**Figure 3:**
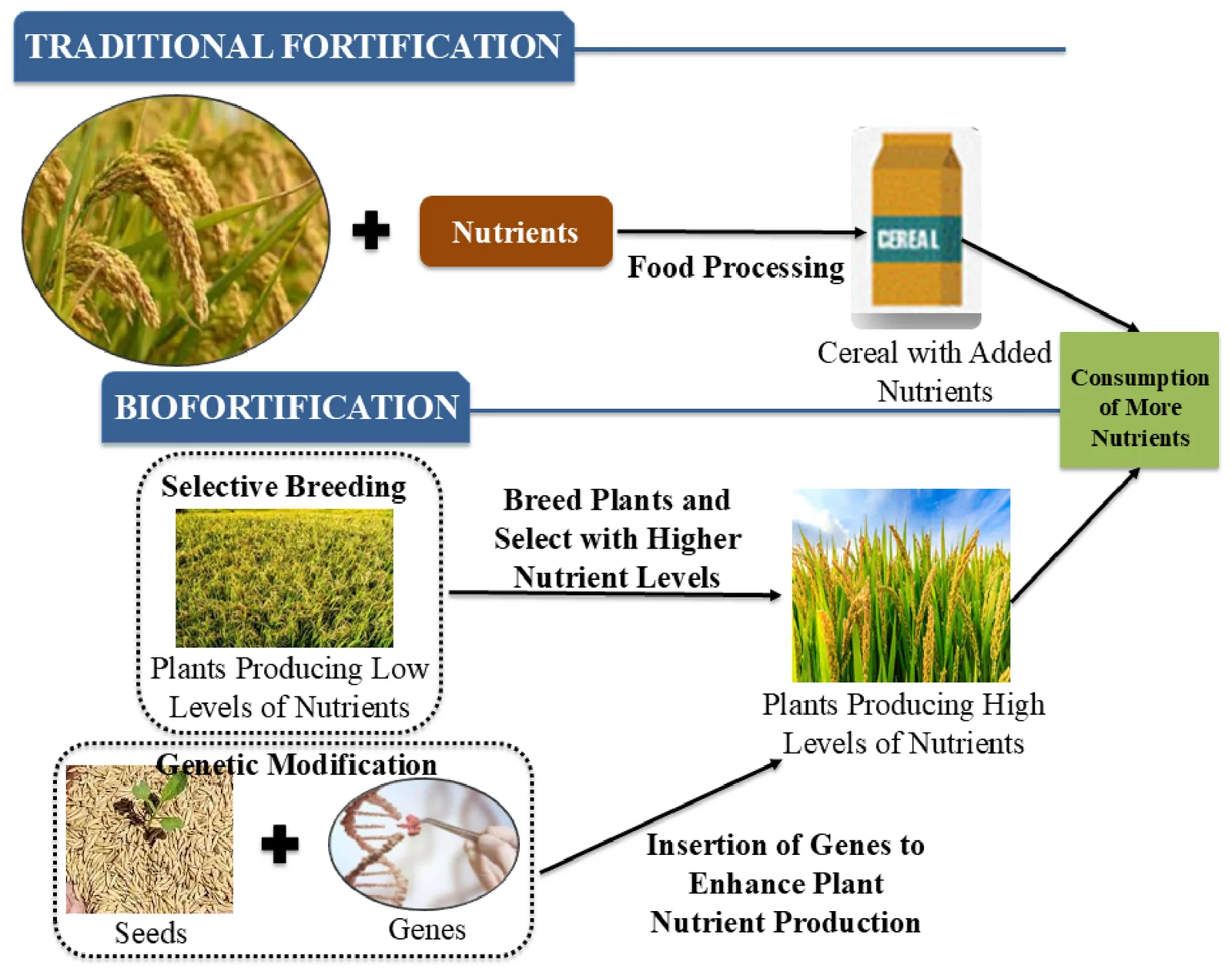
Methods of biofortification for enhancing nutrition to decrease malnutrition.

In nutritional assessment, multiple ways to measure the nutritional status of cardiac patients and assess for risk of malnutrition are available [120]. Specifically, anthropometrics (body measurements and proportions), clinical history, biochemical (lab samples) measurements, a physical exam, and food logs are common approaches for assessment and evaluation purposes [122,123,124,125,126]. Health care professionals can tailor dietary advice that is more effective at preventing or managing diseases by using genomic data, which can predict whether an individual can benefit from specific dietary patterns. A significant shift away from general dietary recommendations to precision nutrition, which combines genomics with other aspects of personal health-related data, is made possible through high-throughput technology that allows identification of responders and non-responders to changes in dietary behaviour. Children’s nutritional health should not be evaluated based only on conventional measures like height and weight, especially if they have neurological impairments (NI). Rather, a more thorough strategy that considers nutritional status and laboratory evaluations is advised [127]. When normal height measures are not possible, regular assessments of tibial length or knee height are recommended for evaluating linear growth. Since blood levels of albumin and pre-albumin do not usually reflect calorie deficits in this population, laboratory parameters such as these may not be a reliable indicator of nutritional status [128]. Given the recognized risk of deficiencies in these parameters, periodic evaluation of micronutrients, including vitamin D, iron, calcium, and phosphorus, is warranted [129]. There is evidence to substantiate that children with NI consume significantly fewer minerals and may put these children at risk for borderline micronutrient status, which requires tracking and evaluation [130]. Recent changes in food formulation for nutritional therapies have shown targeted nutrition, reduced excessive sodium, and added nutrients to agricultural foods [131]. Manufacturers are urged to decrease the sodium content of processed goods and develop spices that preserve flavor while consuming less sodium. To fill specific nutritional gaps among various dietary patterns, like vegetarian and vegan diets, personalized food production is being created [132]. Meeting nutritional needs also heavily depends on improving agricultural methods and practices, such as genetically modifying crops to increase their nutritional content. Another tactic used to enhance food quality and address global hunger and health concerns is the fortification of agricultural goods using nutrient-enriched fertilizers [133]. The technique of producing micronutrient-rich food crops by traditional breeding or genetic modification using contemporary technologies is known as biofortification. For populations with limited access to alternative diets and other micronutrient therapies, this is a practical and economical way to deliver micronutrients. Instead of manually adding nutrients to the food as it is being processed, this type of fortification emphasizes enhancing the nutrient profile of the food crop as it develops and matures. In developing nations where a significant portion of the population is unable to buy enough fruits, vegetables, legumes, fish, and other types of food products that satisfy their basic nutritional needs, biofortification of staple food crops has emerged as a very effective solution to address the issue of a lack of nutrients. Genetic modification and selective breeding are the two main techniques used in the biofortification process (Figure 3). The conventional method, known as selective breeding, involves combining high-yielding cultivars with inherently nutritious crops. Nutritionists must keep an eye on the hybrid types’ development to determine whether consumers can utilize the higher nutritional levels and how the food crop’s processing, storage, and cooking affect these levels. Genetic modification is a technique used by adding foreign genes from the wild crop of the same species or other species that code for greater production of specific nutrients or disease resistance. A crop’s genetic composition can be altered, potentially increasing the host crop’s nutrient content and quality. Alternatively, a crop can be made rich in a range of nutrients by stacking multiple genes that code for different nutrients. Golden rice, which has been enhanced with beta-carotene, a precursor of vitamin A, is one of the most magnificent examples. The techniques discussed above show how it is possible to determine the individual nutritional needs of people and use these needs in formulating fortified food products. However, the real significance of such techniques is best shown in terms of application through examples of personalized fortified foods. The following case studies provide examples of how such needs can be used in designing personalized food products. As shown in Figure 3, there are different methods employed for the improvement of the nutritional value of foods by traditional fortification and biofortification. In traditional fortification, the addition of essential nutrients into food products is done during the processing stage of the food. The process may include the enrichment of the nutrient value of cereal products. Biofortification entails an increase in the nutrient value of crops in the production stage of the crops. The methods include selective breeding and genetic modification of plants to increase the nutrient value. Selective breeding refers to the development of crops with increased concentration of the targeted nutrients, while genetic modification may increase the production or accumulation of the nutrients through genetic modifications.

#### 3.4.2. Case Studies of Personalized Fortified Products

A study by Carey investigates the development and evaluation of an exemplary fruit-based bioactive functional food formulated for consumption in a context of physical activity and exercise [130]. The study used a product development and physiological efficacy testing process along with consumer analysis for the evaluation of the functionality and acceptability of the developed food product. As compared to the following generalized dietary advice, the study highlighted the significance of physiological needs and consumer features while formulating functional foods [134]. Traditional fortification methods involve adding vitamin D to milk, which has been shown to enhance the vitamin D status of the population. However, they often overlook individual differences in factors like age, body weight, exposure to sunlight, diet, and genetics. Studies have proven that individualized dosages, which take into consideration an individual’s unique dose requirements, can improve both the efficacy and safety of the nutrient through personalized nutrition methods. Personalized fortification could potentially overcome some limitations of conventional “one-size-fits-all” fortification by considering individual differences in age, body weight, dietary intake, physiological status, lifestyle, and nutrient requirements. Technologies such as biochemical assessment, dietary history, digital health platforms, ML algorithms, and point-of-care diagnostics may facilitate estimation of individual nutrient requirements and enable adaptive formulation or dosing. Though the study does not examine personalized vitamin D supplementation or personalized vitamin D-enriched beverages, such uses should be seen as possibilities for the future.

Personalization of fortification is likely to solve some of the problems of the traditional approach to fortification by taking into account the variability of individuals in terms of age, body mass, nutrient uptake, physiological state, and lifestyle. Some of the technologies that can help in the process of calculating individual nutrient requirements include biochemical analysis, nutritional history, use of digital health apps, and ML methods and diagnostic devices at point-of-care. At the same time, there is a lack of clinical data indicating the effectiveness of personalized fortified products compared to regular fortification. While pilot studies show higher serum vitamin D retention and customer satisfaction with diversified adaptive system options, despite significantly higher vitamin D fortification products currently available in the marketplace, there are real practical issues to be tackled related to long-term vitamin stability, safe tolerable upper limits of fortification, as well as equality in affordable access. This case study shows that personalized fortification is the natural evolution of functional food from food technology into digital health, and to nutrition science centred on acting on precision nutrition in everyday dietary intake [135]. A study conducted by Borel and his colleagues is centered on the improvement of personalized, rather than generalized, iron fortification of cereals, an important food group, to counteract the population levels of iron deficiency anemia due to individual differences in iron metabolism and absorption because of genetic predisposition [136]. The study considered research data obtained from human trials with regard to genetic variations that influence the mechanisms associated with the absorption, transportation, metabolism, and bioavailability of nutrients. It is emphasized that genetic variations might play a role in causing the variability of individual reactions to nutrients, but the scale of such influences depends on the type of nutrient and the gene involved. The results provide a scientific background for personalization of nutritional strategies based on individual genetic and physiological peculiarities [136]. From these examples, it is evident that personalization in enriched and functional foods can be achieved through variations in physiological needs, diet, genetics, and consumer preferences rather than increasing nutrient concentrations. Well-conducted intervention research is required with an appropriate number of subjects and quantifiable efficacy of benefits to ascertain whether there is a clinical benefit of personalized fortification over traditional population-based fortification. As shown by these examples, personalization is not just about varying the amount of nutrients; there are also differences in genetic make-up, dietary preferences, metabolic rates, and health demands. Personalized fortification takes into account the choice of functional ingredients.

#### 3.4.3. Functional Ingredients’ Role in Personalization

Functional food ingredients such as probiotics, prebiotics, omega-3 fatty acids, and plant bioactive molecules have been studied for possible applications in personalized nutrition. There is evidence that these functional food ingredients are capable of influencing gut microbial populations, metabolite generation, inflammatory signaling pathways, and nutrient metabolism. But one has to distinguish between these biological activities and the actual clinical benefits of these ingredients. For instance, prebiotics are capable of affecting gut microbial populations and increasing short-chain fatty acid (SCFA) production, based on experimental research. The effect on the barrier function and immune signaling pathways is also claimed. However, none of the biological effects mentioned above proves that prebiotic-fortified foods protect against chronic diseases or treat immune conditions [137,138]. Clinical studies in humans appear more inconsistent. While some randomized clinical trials have found positive effects of particular functional foods or diets on certain nutritional or metabolic parameters, others have failed to demonstrate consistent results. It is important to treat claims about the effects of particular foods on improving immunity, fighting inflammation, enhancing metabolism, or preventing disease with caution. Such claims must be limited to outcomes demonstrated in randomized clinical intervention trials in humans [139,140]. Genetic variation might be one of the factors explaining individual variability in nutrient metabolism and absorption. However, data that genotype-based iron fortification offers better clinical outcomes than conventional iron fortification is still lacking. Hence, both genetic data and information on an individual’s microbiome should be considered promising but not validated tools in clinical practice.

A study proved that SCFAs can improve metabolic functioning and serve as a preventative strategy for chronic states of disease; these bioactive compounds are also worth noting in tailored dietary treatments. Personalized methods that consider each person’s genetics and microbiota composition to host and promote health outcomes will be part of the future of nutritional research [141]. In conclusion, research has shown that probiotics, prebiotic fibers, omega-3 fatty acids, and plant-based bioactives offer several health benefits; however, the results may vary depending on social environment, genetics, dosage, and baseline nutritional status. Probiotics may lower the prevalence of respiratory infections in youngsters, according to specific research, whereas vitamin D may improve older persons’ responses to vaccinations [142]. It has been observed that omega-3 fatty acids strengthen the immune system and decrease inflammation. Nevertheless, there is inconsistent evidence regarding these substances among trials, suggesting that interpretation should be done with caution. Individual differences in how each person reacts to these bioactive substances emphasize the value of personalized dietary strategies and imply that specialized therapies based on evaluations of the microbiome and genetic makeup may enhance results [140]. Apart from genetics and metabolism, individual variations in the gut microbiome may affect the metabolism of nutrients and reactions to dietary interventions. Therefore, considering this, the gut microbiome is another level of personalization that needs to be taken into account in the development of fortified foods.

### 3.5. Gut Microbiome and Its Role in Personalized Fortification

The gut microbiome has recently emerged as an important factor involved in nutrient metabolism and individual differences in dietary metabolism. Observational and mechanistic studies, as well as animal models, have shown connections between the composition of the gut microbiome and metabolism, immunity, and digestive system functioning. However, the existence of such a connection does not mean that the effect is causal, and current data on the microbiome are not sufficient to use it as a predictor of disease resistance or response to treatment. Polysaccharides and other compounds that can be fermented by bacteria in the large intestine can affect the composition of the microbiome and levels of SCFAs. Such biological mechanisms exist that would make it possible for the diet to affect the metabolism of the host organism. Nevertheless, there are significant differences between individuals in the impact of such factors, and the evidence on fortified foods is currently insufficient. Several investigations utilizing machine learning methods have shown that microbial features could influence postprandial glycemic response prediction in individuals. While these predictions indicate feasibility and association, not causal proof, metabolism due to the intake of dietary fibers has been correlated with higher levels of Prevotella in human intervention trials; additionally, the mechanism of this effect was validated by transfer experiments with germ-free mice [143]. As these results incorporate both human intervention and animal mechanistic evidence, the information must not be considered as evidence that microbiome manipulation prevents diabetes or any other metabolic diseases. As presented, the study contains the statement about Prevotella and suggests that such an approach is suitable for alleviating diseases. The study divides human intervention evidence from the animal mechanistic evidence. In addition to metabolic disorders, personalized nutrition may be used as a prophylactic treatment for people at higher risk of lifestyle- or blood-related disorders, along with supportive treatment for immune disorders, particularly those involving the gut, cancer, and neurological disorders. The food influences the general function of the intestinal microbiome, particularly the kinds of polysaccharides that are ingested [137]. Polysaccharides are an interesting dietary source with advantageous health qualities due to their lower cost, availability, and relative safety; yet, to benefit human health, the precise concentration of individual polysaccharides or combinations of polysaccharides must be established. Because of the great degree of compositional individuality, gut microbiota manipulation is not simple [144]. Only 14 of the 664 genera that were discovered in a cohort of over 3000 individuals were present in 95% of the individuals [145]. According to Vandeputte et al., the microbiome in the adult gut differs greatly from person to person in terms of both absolute and relative numbers of genera as well as their presence. Both ML and the rational method can be useful when choosing a diet [146]. Microbiome signatures and their metabolic characteristics are recognized for logical design. Beneficial foods for all kinds of microbiome can be found to achieve the intended outcomes once the microbial community has been characterized. Any measurable attribute can be employed with the ML approach, which works best for complicated traits and does not necessitate prior understanding of complex systems [147]. A study was conducted on 800 participants where their blood glucose levels were tracked using an ML technique to determine their postprandial glycemic reactions [147]. Individual differences in glucose metabolism in response to dietary fiber were also observed. According to Kovatcheva-Datchary et al., people whose gut microbiome comprised a higher concentration of the species Prevotella had increased glucose tolerance [143]. These studies highlight the viewpoint of these procedures for choosing a dietary intervention that may be appropriate for any person or group to help ameliorate pathological conditions [143].

#### 3.5.1. Interconnection Between Gut Microbiome and Nutrient Metabolism

The gut microbiome assists in the digestion and metabolism of food components and generates metabolites such as SCFAs, which include acetate, propionate, and butyrate. The effects of SCFAs on the intestinal barrier function, immune signaling, energy metabolism, and gene regulation have been demonstrated in cellular and animal experiments. Mechanistic evidence provides a biological foundation for understanding the relationship between the gut microbiota and the host, but it cannot be considered equivalent to proven clinical effects in humans. The consumption of dietary fiber, microbial population, production of SCFAs, and metabolic consequences have been shown in human studies. However, the strength of the association varies from individual to individual and does not prove that increased production of SCFAs through targeted foods prevents obesity, insulin resistance, diabetes, heart disease, or inflammatory diseases. Moreover, butyrate has been found to affect regulatory T-cell reactions and the barrier function of the intestine. There is a lack of evidence that targeted food interventions based on the gut microbiome lead to clinical-level immune modulation or prevent immune-mediated diseases in humans [137,138]. In summary, SCFA-related mechanisms can be treated as a promising target for biology but not as established therapy or prevention. The future randomized controlled trial needs to assess whether targeted foods result in reliable benefits on clinical outcomes and whether the effect persists for a long time.

SCFAs play an important role in regulating insulin sensitivity and the liver’s utilization of carbohydrates and fats [148,149,150]. Of the SCFAs studied, propionate and acetate are the two most studied, which are produced by gut flora in the colon and subsequently transported to the liver. After propionate reaches the liver, it inhibits fatty acid synthesis, decreasing lipogenesis and serving as an important gluconeogenesis substrate [151,152]. Propionate also has anti-inflammatory properties that are required for decreasing chronic inflammation associated with metabolic diseases [153]. Conversely, acetate aids in preserving a healthy equilibrium between the synthesis of lipids and their utilization as a source of energy. It has a protective role when production is balanced, but when it is present in excess, it may encourage fat synthesis in the liver. Hepatic fat accumulation, a risk factor for non-alcoholic fatty liver disease, must be prevented by preserving this balance. Furthermore, SCFAs alter the expression of genes in the liver that control the inflammatory response and the metabolism of fats and carbohydrates by blocking HDAC. SCFAs in the liver improve insulin sensitivity, control blood sugar, and prevent metabolic liver diseases. Because they control inflammation and the immune response, SCFAs are vital parts of the immune system. One of their most important functions is the suppression of histone deacetylase (HDAC), which permits the epigenetic regulation of immune cell gene expression. This promotes the activation of anti-inflammatory genes and reduces the expression of pro-inflammatory genes to preserve immunological tolerance in the stomach and other body regions and avoid an excess of inflammation that could lead to autoimmune or inflammatory illnesses [154]. Additionally, SCFAs influence G protein-coupled receptors, including FFAR2 and FFAR3, which are present in immune cells such as dendritic cells, neutrophils, and macrophages. When these receptors are activated, pro-inflammatory cytokines like interleukin-6 (IL-6) and tumor necrosis factor-alpha (TNF-α) are released less frequently, and anti-inflammatory cytokines like interleukin-10 (IL-10) are produced more frequently. Maintaining the equilibrium between pro- and anti-inflammatory immune responses depends on this regulation [155].

SCFAs are also protective in chronic inflammatory disorders. For example, butyrate lessens overall immunological activation by maintaining the intestinal barrier due to the stabilization of tight junctions among epithelial cells that prevent bacteria and their products, including lipopolysaccharides (LPS), from entering the circulation. This function is crucial for eliminating chronic inflammation and disorders associated with gut dysbiosis, including metabolic syndrome and inflammatory bowel disease (IBD) [156]. SCFAs also influence the differentiation and activity of regulatory T cells (Tregs), which are a subset of immune cells that play important roles in preventing autoimmunity and modulating exacerbated immune responses. Also, butyrate encourages the growth of Tregs, which helps to maintain immunological tolerance and lower inflammation throughout the body. Because non-ionized SCFAs can break through microbial membranes, dissociate in the cytoplasm, lower intracellular pH, and stop the development of vulnerable bacteria, such as antibiotic-resistant *Enterobacteriaceae*, they can stop the growth of some harmful bacteria [157]. Conclusively, SCFAs have a significant effect on the immune system, assisting in the regulation of inflammation, fortification of the intestinal barrier, and preservation of the immunological balance required to avert autoimmune and inflammatory disorders [158]. *Salmonella enterica*, *Escherichia coli* (AIEC), *Helicobacter pylori*, and *Pseudomonadota* (Proteobacteria) are some potentially harmful bacteria that can stimulate bacterial migration and the immune system. Inflammatory metabolites can be produced by the toxic *B. fragilis* and Bacteroides vulgatus, both of which belong to the phylum Bacteroidota (Bacteroidetes). Toxins produced by *Clostridium difficile* and *Clostridium perfringens*, which belong to the phylum *Bacillota* (Firmicutes), harm the intestinal epithelium. The gut–brain axis, which is the two-way connection between the gut and brain, is also influenced by the gut microbiome. The microbiome stimulates G protein-coupled receptors in the immunological and neurological systems, including FFAR2, FFAR3, GPR109A, and OLFR78, by producing SCFAs. To lessen brain inflammation and support central nervous system homeostasis, these SCFAs alter neuronal activities, control cytokine production, activate microglia, and shield the blood–brain barrier [159,160,161,162,163].

#### 3.5.2. Microbiome-Based Emerging Approaches for Fortification

The gut microbiota can be changed by new treatments that show promise in restoring or changing the composition of the microbiome. A new approach to treating a range of conditions linked with gut dysbiosis is microbiome therapy, which uses modulatory, additive, and subtractive methods in microbiome engineering. The use of natural or engineered microbes, selective antibiotics, bacteriophages, and bacteriocins is a promising treatment for a variety of ailments, from intestinal infections to metabolic and neurological illnesses. Using probiotics or synbiotics that can be native or genetically modified microbes, like *Lactobacillus* and *Bifidobacterium*, to enhance specific functions or address imbalances in the gut microbiome is the aim of additive treatment. Native microorganisms are used to promote digestion, increase immunity, reduce dangerous bacteria, and restore microbial balance when dysbiosis arises [164]. An effective supplemental treatment for irritable bowel syndrome (IBS) is autoprobiotics, which are autochthonous bacteria taken from patients and grown in the lab. These outcomes revealed that additive therapy can result in long-term clinical improvement in IBS therapy, specifically when integrated with tailored probiotics [165]. Subtractive-based treatment utilizes bacteriophages, bacteriocins, and antibiotics to minimize harmful bacteria in the stomach. Because they are intended to exclusively target destructive bacteria without significantly changing the healthy microbiome, selective antibiotics, like rifaximin, are beneficial in conditions like IBS. Bacteriophages, which are viruses that infect and kill certain bacteria, can be used to treat antibiotic-resistant diseases, providing a personalized method to eradicate pathogens without endangering good bacteria [166]. Bacteriocins are antimicrobial peptides produced by some bacteria that prevent rivals from growing and provide a targeted way to eradicate undesirable bacteria without altering the equilibrium of beneficial microorganisms. Intestinal infections and microbiota alteration may benefit from these peptides [167].

Modulatory treatment seeks to alter the composition and function of the microbiota without introducing or eliminating microbes using methods such as diets, prebiotics, and postbiotics. Dietary changes or prebiotic supplements may improve gut health and reduce pathogen proliferation by encouraging the growth of healthy microorganisms such as lactobacilli and bifidobacteria [168]. Moreover, postbiotics—which are thought of as the next generation of probiotics—are extracts free of live bacteria that show modulatory effects on the gut microbiome. Examples of postbiotics that have anti-obesity and anti-diabetic effects include folate, lipopolysaccharides, enzymes, SCFAs, vitamin B12, and K. These mechanisms include increasing energy expenditure, lessening adipocyte formation and differentiation, and regulating gut dysbiosis. Postbiotics are therefore believed to be a useful strategy for treating metabolic problems as well as enhancing gut health [169]. It is feasible to create novel strategies, such as employing native or altered microbes to carry out tasks, such as generating medicinal compounds, improving food digestion, or influencing the immune system. Innovations in biotechnology have made it feasible to develop altered bacteria that can identify and combat illnesses, break down pollutants, or even make medications in the stomach [169]. From intestinal infections to metabolic and neurological illnesses, bacteriophages, bacteriocins, and selective antibiotics are promising treatments [170]. Controlled broad-spectrum antibiotics [171], bacteriophages that target bacteria, and bacteriocins that reduce infections without damaging the rest of the microbiome are emerging treatments that aim to use more selective methods. These methods provide a safer and more accurate means of treating dysbiosis and infections [172,173]. In summary, the use of omics data, personalization of formulations, functional foods, gut microbiota properties, and novel delivery methods constitutes an integrated approach to personalized food fortification. However, the value of such methods ultimately lies in their effectiveness in improving nutritional and health parameters.

### 3.6. Health Outcomes and Clinical Evidence

The level of evidence for the existing food-fortification and personalized nutrition approaches is highly diverse in terms of scientific methodology. Evidence from in vitro experiments and cell cultures mostly includes data about nutrient stability, availability, microbial metabolism, signaling pathways, and controlled release. Evidence from animal studies would give additional information about physiological mechanisms and biological plausibility of the studied phenomena but would not be able to prove human clinical efficacy. Observational studies would allow establishing associations between dietary intake, microbiome features, genetic factors, and health outcomes, but would not be able to show any causal relations. RCTs give stronger evidence of causal effects on particular clinical or biochemical outcomes but still may be limited in terms of sample size, intervention duration, studied population characteristics, and the outcomes analyzed. Population-level fortification programs would give the strongest evidence about the public-health effectiveness of already implemented fortification approaches such as iodine, folic acid, and iron fortification. However, the evidence from those programs should not be extrapolated to personalized fortified foods. Thus, throughout this review, mechanistic and experimental evidence is treated as biological potential evidence, and observational evidence as an association. Moreover, RCT evidence as an intervention effect, and population-level fortification studies as public-health effectiveness evidence.

#### 3.6.1. Evidence-Based Evaluation of Personalized Fortified Foods

Personalized nutrition clinical evidence is becoming more abundant despite there being few clinical trials studying personalized fortified foods. Some recent randomized clinical trials have illustrated the ability of individualized diet recommendations according to metabolism, microbiomes, and health history. For instance, 347 participants in a randomized controlled trial found that personalized diet recommendations based on postprandial glucose and triglyceride responses, microbiomes, and health history. This resulted in lowered triglycerides and helped with weight management, HbA1c, diet quality, and microbiome characteristics as compared to standard dietary recommendations [174]. Similarly, a randomized controlled study conducted over 12 weeks involving 400 overweight or obese Chinese people indicated that personalization of nutrition by taking into consideration anthropometric, phenotypic, genotypic, dietary, and physical activity profiles was more effective in improving BMI, body fat, waist circumference, and several blood biomarkers when compared with general dietary advice [175]. This indicates the possible utility of personalized strategies to tailor nutrient intake according to an individual’s specific profile. Nevertheless, there is very little scientific evidence that evaluates the concept of personalized fortified foods. Hence, further clinical studies are necessary to validate the effectiveness and safety of these foods. For the purpose of context, it is also clear from the evidence gathered through conventional fortification programs that fortification is indeed an effective means of improving micronutrient status, but such results must be taken to support the technique itself rather than personalized fortification.

Global human research has shown that food fortification has a significant impact on vitamin deficiencies. During the 2019 program, vitamin A, iron, and zinc fortification of basic foods enhanced nutrient consumption in disadvantaged communities in Rwanda. In Indonesia, 26% of children’s daily needs for vitamin A were satisfied by cooking oil enriched with the vitamin, reducing the deficiency. For instance, the Philippines found that adding iron to powdered milk was a cost-effective method to address children’s iron deficit, and Costa Rica’s iron fortification program significantly decreased anemia [176]. Since rice is a staple food, it gives a unique chance to correct micronutrient deficits, especially for those involving iron and zinc, using effective fortification approaches [176]. Iron-fortified rice that was distributed through the Public Distribution System in India was well received and decreased anemic outcomes without having any unfavorable side effects [177]. In Brazil, use of ultra-processed foods fortified with micronutrients increased, reducing the overall quality of low-income children’s diets while improving their risk of anemia [178]. Initiatives to fortify food are known to improve public health outcomes and address micronutrient shortages, particularly for vulnerable populations. Furthermore, several extensive studies have shed important light on the effectiveness of fortified meals in practical settings. Energy-protein supplements enriched and given to pregnant and nursing women significantly improved maternal iron status and decreased low birth weight by 19%, according to a randomized controlled trial conducted in Rwanda and Burkina Faso [178]. According to a study conducted in Lebanon, bread supplemented with vitamin D was well received, boosted consumption among low-income households, and had no negative effects on sensory quality [179,180,181]. These results place food fortification at the forefront as a practical and successful strategy to provide micronutrient alleviation to a wide range of people. However, in several low- and middle-income countries, long-term follow-up research assessing biochemical results, adherence, and prolonged effects has not yet been carried out. Table 1 outlines key studies that evaluate how well fortified foods work to correct vitamin deficits and enhance health.

#### 3.6.2. Ethical, Regulatory, and Safety Considerations

Ethical and safety considerations play a vital role in the use of controlled release systems (CRS) and their development in food fortification. This technology ensures safety, transparency, and informed choice for the consumer, particularly when novel materials like nanocarriers or biopolymers are used. Ethical concerns raise potential long-term health impacts, labelling accuracy, and equitable access to fortified foods across various socioeconomic groups. Moreover, regulatory frameworks, like the FDA, EFSA, and FSSAI, demand rigorous evaluation of the stability, toxicity, and biological availability of encapsulated nutrients before the market approval process. Safety assessments mainly focus on interactions among encapsulants and food matrices, degradation behavior in the gastrointestinal tract, as well as the risk of bioaccumulation or allergenicity. Hence, robust ethical oversight and harmonized international regulations are essential to balance innovation with public health protection and consumer trust. Despite the recent support for personalized fortification and nutrient delivery from evidence, transferring such methods from laboratory conditions to mass application in food is difficult. It is necessary to pay attention not only to the nutritional benefits that such methods offer but also to various problems associated with technology, costs, safety, regulation, etc.

## 4. Challenges and Future Directions

Despite the important potential of CRS in food fortification, many challenges hinder its production on a large scale. Technical limitations like sustaining nutrient stability during processing, confirming uniform encapsulation, and attaining precise release kinetics under physiological circumstances are still major hurdles. Economic constraints related to production costs and scalability also limit their widespread usage in low- and middle-income markets. Moreover, gaps in regulatory precision, consumer acceptance, and standardized testing procedures pose barriers to commercialization. Future directions focus on developing cost-effective, biodegradable, and food-grade delivery systems by employing natural polymers and nanomaterials, combining AI and computational modelling for release optimization, and establishing global guidelines for safety and efficacy assessment. Advanced technologies from laboratory innovation to sustainable, consumer-trusted food fortification solutions.

## 5. Conclusions

In conclusion, encapsulation and CRS show a transformative advancement in food fortification, efficiently bridging the gap between food science and nutrition. Moreover, these technologies increase the stability, bioavailability, and target-based delivery of sensitive nutrients like vitamins, minerals, and biologically active compounds, ensuring their functionality throughout processing, storage, and assimilation. By allowing precise nutrient release and minimizing sensory modifications, they offer promising solutions to global nutritional deficiencies and support the development of next-generation functional foods. In addition, the combination of encapsulation technology, CRS, omics approaches, personalized nutrition, gut microbiome-based techniques, AI, and 3D/4D food printing offers possibilities to develop tailor-made fortified foods to cater to the individual nutritional needs of consumers. There are still several issues regarding scalability, safety concerns, compliance with regulations, and production costs, as well as stabilization of nutrients and consumer acceptability that need to be addressed. Therefore, future research in this area needs to concentrate on enhancing the efficiency of encapsulation and CRS, developing affordable and sustainable food-grade delivery systems, carrying out clinical trials on personalized fortified foods, and creating standardized guidelines. Eventually, the integration of innovative encapsulation techniques with personalized nutrition approaches will pave the way for safe, more effective, and sustainable fortified food systems that align with the goals of personalized nutrition and public health improvement.

## Figures and Tables

**Table 1:** Summary of existing scientific evidence on fortified food effectiveness.

Nutrient/Fortification Strategy	Target Population	Key Outcomes Assessed	Evidence of Effectiveness	References
Iron-fortified flour and staple foods	General population, women, and children in LMICs	Reduced iron deficiency and some reductions in anemia; haemoglobin and ferritin improvements were variable by setting.	Fortification generally improves iron status and hemoglobin concentrations; however, reductions in anemia are less consistent across populations and settings.	[182]
Folic acid-fortified flour and cereals	Women of reproductive age; periconceptional period	Substantial reduction in neural tube defects (NTDs) at the population level	Strong population-level evidence demonstrates that folic acid fortification substantially reduces the prevalence of NTDs and improves folate status	[183]
Iodine salt	The entire population, children, and pregnant women were prioritized	Improved urinary iodine, reduced goiter prevalence, and reductions in severe iodine-deficiency disorders	Salt iodization effectively improves iodine status and contributes to the prevention and control of iodine-deficiency disorders at the population level	[184]
Vitamin A-fortified foods	Children and populations with vitamin A deficiency	Improved vitamin A status; variable impacts on morbidity/mortality depending on baseline deficiency and program scale	Fortification can improve vitamin A status and reduce deficiency, although effects on morbidity and mortality vary according to baseline deficiency and program coverage	[185]
Multiple-micronutrient fortification	Population-level (urban/rural)	Increased micronutrient intakes; population reach varies; potential reductions in deficiency indicators	Large-scale multiple-micronutrient fortification can increase micronutrient intake and improve selected nutritional biomarkers, but population-level effects vary between programs.	[186,187]

## Data Availability

No new data were created or analysed in this study. Data sharing does not apply to this article.
